# Supplementary material for: Splice-Junction-Based Mapping of Alternative Isoforms in the Human Proteome
Source: Cell Rep. Author manuscript; Available in PMC 2020 Jan 15. (PMC6961840; doi:10.1016/j.celrep.2019.11.026)
Supplement: 2 [file NIHMS1546469-supplement-2.zip › DF1/PXD009737/TestisTrypsin/TestisTrypsin_10_EIF4G1_APQSTGPPPAPSPGLPQHFYPSR.pdf]

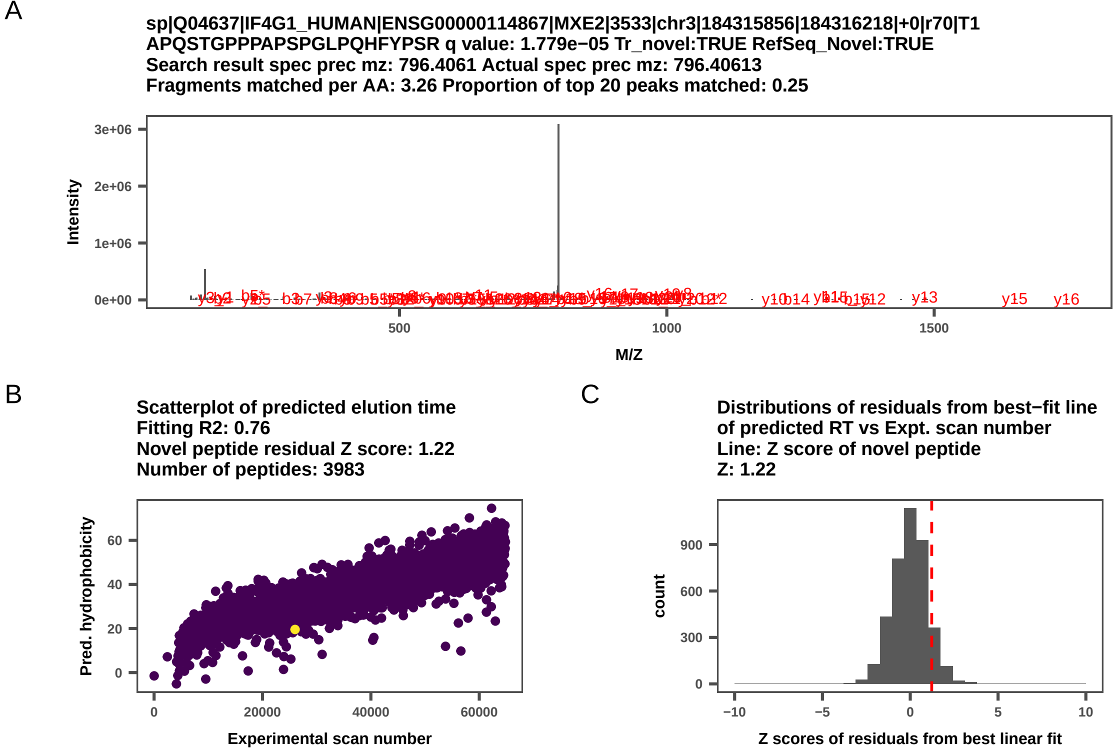
A
sp|Q04637|IF4G1_HUMAN|ENSG00000114867|MXE2|3533|chr3|184315856|184316218|+0|r70|T1
APQSTGPPPAPSPGLPQHFYPSR q value: 1.779e−05 Tr_novel:TRUE RefSeq_Novel:TRUE
Search result spec prec mz: 796.4061 Actual spec prec mz: 796.40613
Fragments matched per AA: 3.26 Proportion of top 20 peaks matched: 0.25
Intensity
3e+06
2e+06
1e+06
0e+00
y10
b14
y13
y15
y16
500
1000
1500
M/Z
B
Scatterplot of predicted elution time
Fitting R2: 0.76
Novel peptide residual Z score: 1.22
Number of peptides: 3983
Pred. hydrophobicity
60
40
20
0
0
20000
40000
60000
Experimental scan number
C
Distributions of residuals from best−fit line
of predicted RT vs Expt. scan number
Line: Z score of novel peptide
Z: 1.22
count
900
600
300
0
−10
−5
0
5
10
Z scores of residuals from best linear fit
